# Supplementary material for: Resolving the full spectrum of human genome variation using Linked-Reads
Source: Genome Res. 2019 Apr;29(4):635–45. doi: 10.1101/gr.234443.118 (PMC6442396; doi:10.1101/gr.234443.118)
Supplement: Supplemental Material [file supp_gr.234443.118_Supplemental_longranger-2.2.zip › longranger-2.2.tar.bz2!/lr2.2-public/lib/stan/cmdstan/stan/doc/api/html/classstan_1_1io_1_1dump.html]

Stan: stan::io::dump Class Reference


|  |  |
| --- | --- |
|  | Stan  2.5.0  probability, sampling & optimization |


- Main Page
- Related Pages
- Namespaces
- Classes
- Files

- Class List
- Class Index
- Class Hierarchy
- Class Members

All Classes Namespaces Files Functions Variables Typedefs Enumerations Enumerator Friends Macros Pages

- stan
- io
- dump

Public Member Functions |
List of all members

stan::io::dump Class Reference

Represents named arrays with dimensions.
More...

`#include <dump.hpp>`

Inheritance diagram for stan::io::dump:

|  |  |
| --- | --- |
| Public Member Functions | |
|  | dump (std::istream &in) |
|  | Construct a dump object from the specified input stream. More... |
|  | |
| bool | contains\_r (const std::string &name) const |
|  | Return `true` if this dump contains the specified variable name is defined. More... |
|  | |
| bool | contains\_i (const std::string &name) const |
|  | Return `true` if this dump contains an integer valued array with the specified name. More... |
|  | |
| std::vector< double > | vals\_r (const std::string &name) const |
|  | Return the double values for the variable with the specified name or null. More... |
|  | |
| std::vector< size\_t > | dims\_r (const std::string &name) const |
|  | Return the dimensions for the double variable with the specified name. More... |
|  | |
| std::vector< int > | vals\_i (const std::string &name) const |
|  | Return the integer values for the variable with the specified name. More... |
|  | |
| std::vector< size\_t > | dims\_i (const std::string &name) const |
|  | Return the dimensions for the integer variable with the specified name. More... |
|  | |
| virtual void | names\_r (std::vector< std::string > &names) const |
|  | Return a list of the names of the floating point variables in the dump. More... |
|  | |
| virtual void | names\_i (std::vector< std::string > &names) const |
|  | Return a list of the names of the integer variables in the dump. More... |
|  | |
| bool | remove (const std::string &name) |
|  | Remove variable from the object. More... |
|  | |
| Public Member Functions inherited from stan::io::var\_context | |
| virtual | ~var\_context () |
|  | |
| void | add\_vec (std::stringstream &msg, const std::vector< size\_t > &dims) const |
|  | |
| void | validate\_dims (const std::string &stage, const std::string &name, const std::string &base\_type, const std::vector< size\_t > &dims\_declared) const |
|  | |

|  |  |
| --- | --- |
| Additional Inherited Members | |
| Static Public Member Functions inherited from stan::io::var\_context | |
| static std::vector< size\_t > | to\_vec () |
|  | |
| static std::vector< size\_t > | to\_vec (size\_t n1) |
|  | |
| static std::vector< size\_t > | to\_vec (size\_t n1, size\_t n2) |
|  | |
| static std::vector< size\_t > | to\_vec (size\_t n1, size\_t n2, size\_t n3) |
|  | |
| static std::vector< size\_t > | to\_vec (size\_t n1, size\_t n2, size\_t n3, size\_t n4) |
|  | |
| static std::vector< size\_t > | to\_vec (size\_t n1, size\_t n2, size\_t n3, size\_t n4, size\_t n5) |
|  | |
| static std::vector< size\_t > | to\_vec (size\_t n1, size\_t n2, size\_t n3, size\_t n4, size\_t n5, size\_t n6) |
|  | |
| static std::vector< size\_t > | to\_vec (size\_t n1, size\_t n2, size\_t n3, size\_t n4, size\_t n5, size\_t n6, size\_t n7) |
|  | |
| static std::vector< size\_t > | to\_vec (size\_t n1, size\_t n2, size\_t n3, size\_t n4, size\_t n5, size\_t n6, size\_t n7, size\_t n8) |
|  | |

## Detailed Description

Represents named arrays with dimensions.

A dump object represents a dump of named arrays with dimensions. The arrays may have any dimensionality. The values for an array are typed to double or int.

See `dump_reader` for more information on the format.

Dump objects are created from reading dump files from an input stream.

The dimensions and values of variables may be accessed by name.

Definition at line 902 of file dump.hpp.

## Constructor & Destructor Documentation

|  |  |  |  |  |  |
| --- | --- | --- | --- | --- | --- |
| stan::io::dump::dump | ( | std::istream & | *in* | ) |  |

inline

Construct a dump object from the specified input stream.

**Warning:** This method does not close the input stream.

Parameters
:   |  |  |
    | --- | --- |
    | in | Input stream from which to read. |

Definition at line 934 of file dump.hpp.

## Member Function Documentation

|  |  |  |  |  |  |
| --- | --- | --- | --- | --- | --- |
| bool stan::io::dump::contains\_i | ( | const std::string & | *name* | ) | const |

inlinevirtual

Return `true` if this dump contains an integer valued array with the specified name.

Parameters
:   |  |  |
    | --- | --- |
    | name | Variable name to test. |

Returns
:   `true` if the variable name has an integer array value.

Implements stan::io::var\_context.

Definition at line 972 of file dump.hpp.

|  |  |  |  |  |  |
| --- | --- | --- | --- | --- | --- |
| bool stan::io::dump::contains\_r | ( | const std::string & | *name* | ) | const |

inlinevirtual

Return `true` if this dump contains the specified variable name is defined.

This method returns `true` even if the values are all integers.

Parameters
:   |  |  |
    | --- | --- |
    | name | Variable name to test. |

Returns
:   `true` if the variable exists.

Implements stan::io::var\_context.

Definition at line 960 of file dump.hpp.

|  |  |  |  |  |  |
| --- | --- | --- | --- | --- | --- |
| std::vector<size\_t> stan::io::dump::dims\_i | ( | const std::string & | *name* | ) | const |

inlinevirtual

Return the dimensions for the integer variable with the specified name.

Parameters
:   |  |  |
    | --- | --- |
    | name | Name of variable. |

Returns
:   Dimensions of variable.

Implements stan::io::var\_context.

Definition at line 1034 of file dump.hpp.

|  |  |  |  |  |  |
| --- | --- | --- | --- | --- | --- |
| std::vector<size\_t> stan::io::dump::dims\_r | ( | const std::string & | *name* | ) | const |

inlinevirtual

Return the dimensions for the double variable with the specified name.

Parameters
:   |  |  |
    | --- | --- |
    | name | Name of variable. |

Returns
:   Dimensions of variable.

Implements stan::io::var\_context.

Definition at line 1004 of file dump.hpp.

|  |  |  |  |  |  |
| --- | --- | --- | --- | --- | --- |
| virtual void stan::io::dump::names\_i | ( | std::vector< std::string > & | *names* | ) | const |

inlinevirtual

Return a list of the names of the integer variables in the dump.

Parameters
:   |  |  |
    | --- | --- |
    | names | Vector to store the list of names in. |

Implements stan::io::var\_context.

Definition at line 1063 of file dump.hpp.

|  |  |  |  |  |  |
| --- | --- | --- | --- | --- | --- |
| virtual void stan::io::dump::names\_r | ( | std::vector< std::string > & | *names* | ) | const |

inlinevirtual

Return a list of the names of the floating point variables in the dump.

Parameters
:   |  |  |
    | --- | --- |
    | names | Vector to store the list of names in. |

Implements stan::io::var\_context.

Definition at line 1047 of file dump.hpp.

|  |  |  |  |  |  |
| --- | --- | --- | --- | --- | --- |
| bool stan::io::dump::remove | ( | const std::string & | *name* | ) |  |

inline

Remove variable from the object.

Parameters
:   |  |  |
    | --- | --- |
    | name | Name of the variable to remove. |

Returns
:   If variable is removed returns `true`, else returns `false`.

Definition at line 1080 of file dump.hpp.

|  |  |  |  |  |  |
| --- | --- | --- | --- | --- | --- |
| std::vector<int> stan::io::dump::vals\_i | ( | const std::string & | *name* | ) | const |

inlinevirtual

Return the integer values for the variable with the specified name.

Parameters
:   |  |  |
    | --- | --- |
    | name | Name of variable. |

Returns
:   Values.

Implements stan::io::var\_context.

Definition at line 1020 of file dump.hpp.

|  |  |  |  |  |  |
| --- | --- | --- | --- | --- | --- |
| std::vector<double> stan::io::dump::vals\_r | ( | const std::string & | *name* | ) | const |

inlinevirtual

Return the double values for the variable with the specified name or null.

Parameters
:   |  |  |
    | --- | --- |
    | name | Name of variable. |

Returns
:   Values of variable.

Implements stan::io::var\_context.

Definition at line 983 of file dump.hpp.

---

The documentation for this class was generated from the following file:

- src/stan/io/dump.hpp


---

[ Stan Home Page ]

*© 2011–2014,
Stan Development Team.*
